# Developmental pathway from child maltreatment to children’s bullying: sex difference in the longitudinal dual-process model of self-esteem and depression

**DOI:** 10.3389/fpsyg.2025.1669772

**Published:** 2025-11-07

**Authors:** Xiangfei Duan, Xianglan Zhang, Yanxin Song

**Affiliations:** 1 College of Education, Ludong University, Yantai, China; 2 Institute for Education and Treatment of Problematic Youth, Ludong University, Yantai, China; 3 Youth Guidance Department, Myongji University, Seoul, Republic of Korea

**Keywords:** child maltreatment, bullying, self-esteem, depression, children

## Abstract

**Background:**

Bullying is potentially linked to childhood maltreatment, yet distinctions among maltreatment types and their underlying mechanisms remain under-examined.

**Objective:**

This study tested whether emotional and physical maltreatment predict children’s bullying perpetration through self-esteem and depressive symptoms, and whether these indirect pathways differ by gender.

**Method:**

In a three-wave longitudinal design spanning approximately 12 months, data were collected at baseline (T1, mid-December, *n* = 780), 6-month follow-up (T2, mid-June, *n* = 774), and 12-month follow-up (T3, mid-December, *n* = 706). Mediation models examined self-esteem and depression as parallel mediators between emotional/physical maltreatment (T1) and bullying (T3). Multi-group analyses and Wald tests compared path coefficients and indirect effects across boys and girls.

**Results:**

Longitudinal mediation revealed that the indirect effect of T1 emotional maltreatment on T3 bullying via T2 self-esteem (full mediation, *β*_boys_ = 0.017, SE _boys_ = 0.008, 95% CI [0.002, 0.032]) and the indirect effect of T1 physical maltreatment on T3 bullying via T2 depression (partial mediation, *β*_boys_ = 0.029, SE _boys_ = 0.013, 95% CI [0.005, 0.053]) were significant only among boys.

**Conclusion:**

The finding that distinct maltreatment types are linked to bullying through different, gender-specific psychological pathways highlights the need for tailored prevention strategies that account for both maltreatment type and gender.

## 1 Introduction

Bullying is defined as one or more students repeatedly directing negative actions toward another student and is characterized by three core features: a power imbalance, the intent to harm, and repetition (Olweus, 1993; Sharp et al., 2002). It is associated with numerous adverse outcomes, including serious emotional and behavioral disorders (Khamis, 2015; UNESCO, 2019). Nevertheless, recent studies show that bullying remains highly prevalent worldwide and its incidence is still increasing (Özcan et al., 2024). Consequently, identifying the risk factors and mechanisms underlying bullying has become a critical research priority, essential for developing effective intervention strategies to reduce its occurrence.

It should be noted that although bullying is a global concern, its concrete manifestations, antecedents, and mechanisms are deeply embedded in specific cultural contexts and social environments (Smith et al., 2019). The present study therefore focuses on bullying among Chinese children. First, China is one of the countries with the highest prevalence of bullying: roughly 20% of primary- and middle-school students experience school-based bullying (as perpetrators, victims, or bully-victims) at least twice a month (Zhang, 2021). Second, the bulk of extant research has been conducted in Western societies; only recently has the literature on Chinese samples begun to expand (Smith, 2023). Yet many Western-derived conclusions may not generalize across cultures (Chung, 2017). China’s “relation-based” collectivism—where individuals are tied to the group through affective bonds rooted in kinship and prolonged co-residence (Hwang, 1987; Zheng and Tian, 2024)—is likely to generate distinct bullying pathways (Migliaccio and Raskauskas, 2016). In this cultural frame, early family experiences are disproportionately influential in shaping later behavior (Huang and Bi, 2025). Accordingly, the current study examines how intra-familial adversities—specifically childhood physical and emotional maltreatment—impact bullying perpetration through a culturally specific mechanism among Chinese children.

### 1.1 Child physical and emotional maltreatment and bullying

Although the cycle-of-violence thesis has long proposed that maltreated children are at heightened risk of perpetrating violence later in life, it offers limited insight into the precise mechanisms underlying this linkage (Widom, 1989). Likewise, empirical studies examining associations between child physical and emotional maltreatment and bullying behaviors have typically treated these two forms of maltreatment as core indicators of childhood adversity, repeatedly documenting their overall relations with bullying (Türk et al., 2021; Merrin et al., 2024), yet rarely unpacking the distinct psychological pathways through which each form of maltreatment exerts its influence. Different types of maltreatment, however, differ markedly in their manifestations and psychological sequelae (Wright, 2007), underscoring the need to disentangle their unique effects—especially for physical and emotional maltreatment, the two most prevalent forms documented worldwide (World Health Organization, 2024). Aetiologically and phenomenologically, the two diverge in essence: emotional maltreatment inflicts psychological trauma primarily through hostile or rejecting acts (Glaser, 2002), whereas physical maltreatment entails bodily harm via hitting, kicking, or other aggressive behaviors (Christian et al., 2015). Building on these distinctions, the present longitudinal study separately examines the prospective links between emotional maltreatment and physical maltreatment, on the one hand, and bullying perpetration on the other. We first hypothesize that both emotional maltreatment and physical maltreatment will positively predict subsequent bullying behaviors.

Notably, a small but growing body of research has begun to highlight the importance of investigating the unique mechanisms linking each maltreatment type to bullying, yielding initial insights. For example, Zhang et al. (2025) employed a two-wave longitudinal design and found that perceived social support and self-compassion significantly mediated the effect of emotional maltreatment on adolescent bullying, with the pathways further moderated by gender. Yet, this study captured only short-term causal ordering and left unexplored a range of additional potential mediators—such as self-esteem and depression—that might also account for the maltreatment-to-bullying link. Leveraging three-site longitudinal data, the current investigation therefore extends prior work by testing further mediating mechanisms (and their gender specificity) that may underlie the longitudinal associations between emotional/physical maltreatment and bullying perpetration.

### 1.2 The mediating role of self-esteem

Self-esteem—an individual’s global evaluation of self-worth (Zhang H. et al., 2022)—encompasses basic beliefs about one’s competencies and value (Cast and Burke, 2002). Attachment theory posits that children construct internal working models of the self and others through early caregiver interactions (Bowlby, 1969). Chronic emotional or physical maltreatment embeds denigration and corporal punishment within these interactions, leading maltreated children to form negative self-representations—i.e., low self-esteem. The hierarchy-of-adaptation perspective on defense further argues that individuals with diminished self-esteem are more likely to rely on immature defenses, including bullying (Cramer and Jones, 2008; Giovazolias et al., 2017). Self-esteem is therefore expected to mediate the path from both emotional and physical maltreatment to bullying perpetration.

Importantly, although both maltreatment types are maltreatment, their routes via self-esteem may differ. A recent meta-analysis revealed a weak association between physical maltreatment and self-esteem, whereas emotional maltreatment showed a moderate negative relation (Zhang H. et al., 2022). Persistent devaluation and rejection, the hallmarks of emotional maltreatment, may more directly erode self-worth (Chen and Qin, 2020), generate stronger feelings of low self-esteem, and prompt individuals to restore a sense of dominance or value through bullying (Laninga-Wijnen et al., 2019). Thus, the indirect effect of emotional maltreatment on bullying through self-esteem is anticipated to be stronger. In addition, research indicates that male adolescents are more prone to exhibit low self-esteem and corresponding externalizing problems following maltreatment, suggesting gender-specific vulnerability (Wan and Xu, 2022). The present study will therefore examine whether this mediated pathway varies by gender, with boys showing a heightened risk of self-esteem deficits and heightened bullying propensity after maltreatment.

### 1.3 The mediating role of depression

Depression—an affective disorder marked by persistent sadness, loss of interest, appetite and sleep disturbances, slowed thinking, and poor concentration (Miret et al., 2013)—is a well-documented sequela of child maltreatment (Berber Çelik and Odacı, 2020). As an internalizing condition, depression undermines adaptive emotion regulation and erodes interpersonal trust, leading some youth to misuse bullying as a maladaptive outlet for inner pain or as a tool to forge social connections (Cooley et al., 2019). Empirical work further shows that higher depressive symptoms predict greater likelihood of bullying perpetration (Liu et al., 2022). Thus, depression is expected to mediate the pathway from both physical and emotional maltreatment to bullying.

More specifically, regarding physical maltreatment, the post-traumatic stress model (Ehlers and Clark, 2000) posits that physical maltreatment, as a traumatic event, disrupts emotion-regulation capacities and shatters core beliefs about the self and the world, generating persistent helplessness, worthlessness, and emotional distress—cardinal features of depression. This maltreatment-induced depressive state, in turn, increases the probability of engaging in bullying behaviors (Holt et al., 2009). With respect to emotional maltreatment, hostile or rejecting caregiver behavior impairs emotional regulation and distorts self-cognition, fostering chronic internalizing symptoms such as depression. These symptoms reduce social competence and raise the likelihood that the youth will aggress against others to gain a sense of control or to discharge negative affect (Li et al., 2025). Moreover, the indirect pathway from maltreatment to bullying via depression may differ by gender. Owing to traditional gender socialization, boys are more strongly socialized than girls to appear “tough” and to “cope alone”; consequently, they feel greater shame about openly expressing maltreatment-related distress or seeking help (Addis and Mahalik, 2003). When this pain remains unexpressed, it is more likely to crystallize into depressive symptoms, which may then be displaced into bullying others as a maladaptive outlet.

### 1.4 The chain mediating role of self-esteem and depression

According to attachment theory (Bowlby, 1969), the formation of self-esteem is rooted in early interactions with primary caregivers. A supportive family climate fosters secure attachment and, in turn, high self-esteem, whereas children reared in contexts of emotional neglect or abuse fail to develop such security and are prone to low self-esteem (Oshri et al., 2017). The vulnerability model further proposes that chronically low self-esteem, as a stable trait, constitutes a key diathesis for depression (Klein et al., 2011). Meta-analytic evidence (Sowislo and Orth, 2013) corroborates this view, showing a robust effect of low self-esteem on subsequent depressive symptoms. Depression, in turn, is a well-established risk factor for bullying perpetration (Dutton and Karakanta, 2013); afflicted youths, overwhelmed by negative affect, may victimize weaker peers to gain a fleeting sense of mastery and power that momentarily counteracts their own helplessness. Building on these links, the present study first examines self-esteem and depression as separate mediators of emotional and physical maltreatment on bullying, and then tests the chained sequence “maltreatment → low self-esteem → depression → bullying.” We hypothesize that this chain will be significant for both maltreatment forms.

### 1.5 The current study

Although the cycle-of-violence framework (Widom, 1989) is well established, the mechanisms that distinguish the effects of emotional from physical maltreatment remain under-explored: (1) whether each maltreatment type (emotional vs. physical) prospectively predicts bullying in a representative child sample; (2) whether self-esteem and depression operate as mediators; (3) whether the mediating pathways from emotional/physical maltreatment to bullying through self-esteem or depression differ by gender; (4) whether a chained mediation (maltreatment → self-esteem → depression → bullying) exists for both maltreatment forms. By integrating findings across these aims, we aim to clarify the maltreatment-specific psychological pathways that give rise to bullying, thereby informing the development of targeted interventions.

The following hypotheses will be proposed in this study. H1: Both emotional and physical child maltreatment will positively predict later bullying perpetration; H2: Self-esteem will mediate the association between emotional/physical maltreatment and bullying; H3: Depression will mediate the association between emotional/physical maltreatment and bullying; H4: The indirect paths from emotional/physical maltreatment to bullying via self-esteem or depression will differ by gender; H5: A chained indirect effect (maltreatment → self-esteem → depression → bullying) will emerge for both emotional and physical maltreatment.

## 2 Method

### 2.1 Participants

**Figure 1:**
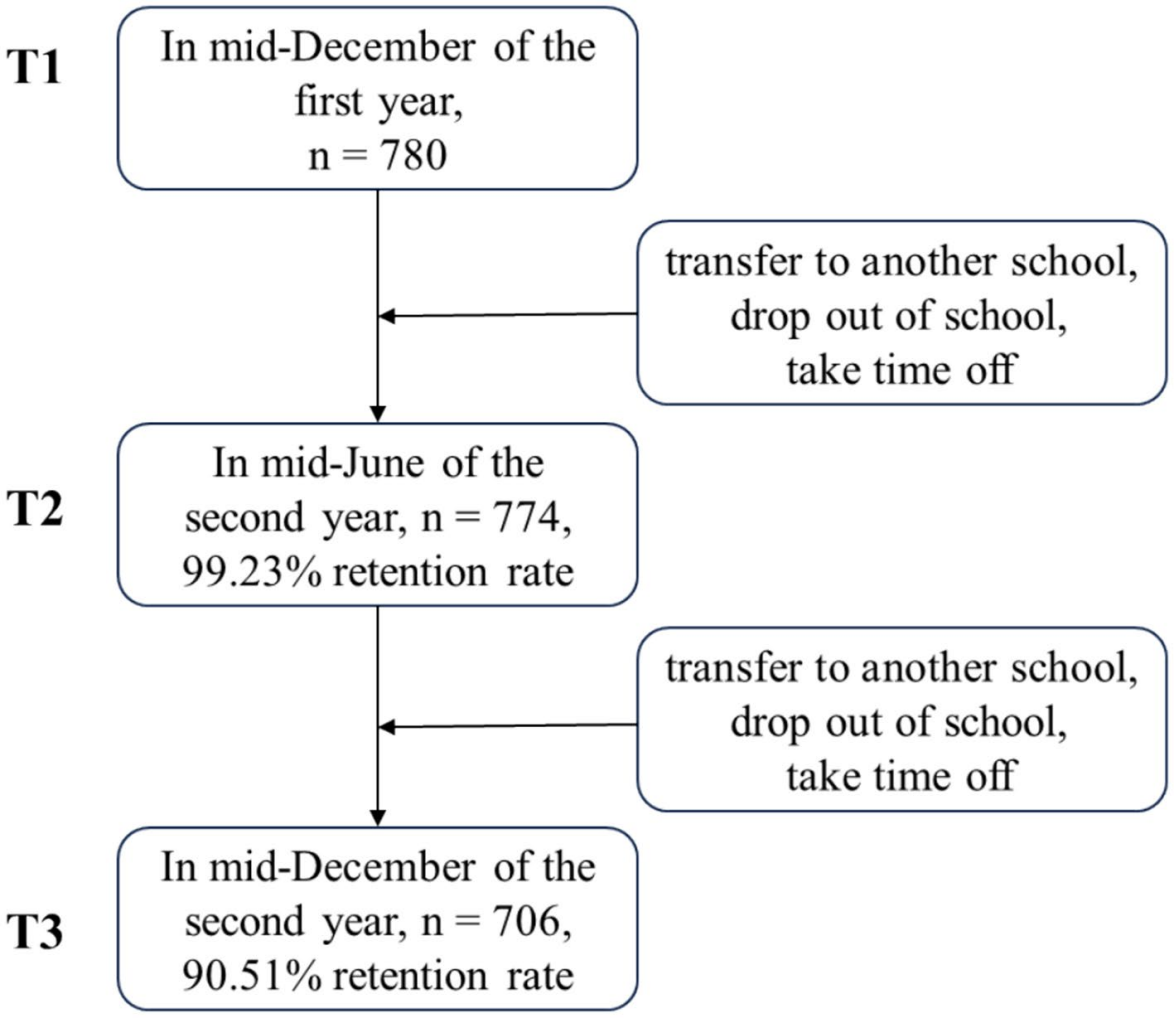
Participant recruitment flowchart.

Participants were recruited from 20 classes across three primary schools—two urban and one rural—located in Tangshan, Hebei Province, China. We collected data on child maltreatment in mid-December of the first year (T1), self-esteem and depression in mid-June of the second year (T2), and child bullying in mid-December of the second year (T3). The key variables were assessed at different waves to establish temporal precedence between predictors, mediators, and outcomes, thereby strengthening the inference of causal pathways and reducing potential common method bias. The valid data were obtained from 780 individuals at T1, 774 at T2, and 706 at T3. Participant attrition during the follow-up period was primarily due to school transfers, drop out of school, and absence on the day of survey administration (see Figure 1). There were 487 males, accounting for 62.4% of the sample at T1, with an average age of 9.02 years (SD = 0.60). Subjective socio-economic status (subjective SES) was assessed by self-reported and about 1.7% of students were high subjective SES, 10.5% were middle-high, 61.9% were middle, 20.1% were middle-low, 4.7% were low, and 1.0% were missing for this variable. For data collection, the consent of school teachers and parents were obtained in advance. Questionnaires were distributed to each class individually. Before the test, trained research assistants explained the purpose and significance of data collection, in addition, they provided instructions for filling out the survey. Anonymity and confidentiality were ensured for participants.

### 2.2 Measures

#### 2.2.1 Childhood trauma questionnaire (CTQ)

Child maltreatment was measured by the CTQ. A total of 25 items makes up the CTQ (Bernstein et al., 2003), and there are five dimensions: emotional/physical/sexual abuse, emotional/physical neglect. Five items make up each dimension, and there are five levels of scoring for each item, with 1 indicating “not at all” and 5 indicating “always.” It has been demonstrated that the CTQ has a high level of reliability and validity (Zhang R. et al., 2022). The current study only measured emotional/physical abuse, emotional/physical neglect. Due to our research objectives and the potential impact of answering questions about sexual abuse on students, we did not use the sexual abuse subscale. Following the approach of Iffland et al. (2012), composite variables were created. “Emotional maltreatment” combines scores from the CTQ emotional abuse and emotional neglect subscales, and “physical maltreatment” combines scores from the physical abuse and physical neglect subscales. After reverse-scoring the 7 items, sum the scores in each dimension, with higher scores indicating more severe abuse. The Cronbach’*α* coefficient of the scale was 0.90, the Cronbach’*α* coefficient of the emotional/physical abuse, emotional/physical neglect were 0.86–0.91.

#### 2.2.2 Self-esteem scale (SES)

In 1965, Rosenberg compiled the SES (Rosenberg, 1965) to measure feelings of self-acceptance and self-worth among children. The scale has 10 items in total (e.g., I feel that I am a valuable person, at least on par with others.) and adopts 4-point Likert’s scoring method from 1 (strongly disagree) to 4 (strongly agree). There are four reverse scoring questions. The self-esteem of those with higher scores is higher. The SES possesses good validity and reliability (Zhou et al., 2018; Wood et al., 2021). The Cronbach’*α* coefficient of the scale was 0.85.

#### 2.2.3 Center for epidemiological studies depression (CES-D) scale

The Chinese version of the CES-D scale based on Radloff (1977) was used to measure individual depression in this study (Chen et al., 2009). The time range of the scale test is “In the past week.” Twenty items are included in the scale (e.g., I did not feel like eating; my appetite was poor), and items are rated from 0 (not at all) to 3 (a lot). There are 4 items that require reverse scoring. After reverse-scoring these items, sum the scores of all items, with higher total scores indicating more severe depressive symptoms. Previous studies have demonstrated that the CES-D scale is suitable for children and children aged 8 and above (Tatar et al., 2013). The Cronbach’*α* coefficient of the scale was 0.90.

#### 2.2.4 Bully/victim questionnaire (BVQ)

This study used the bullying subscale from the Chinese version of Olweus’ Children’s Bullying Questionnaire, revised by Zhang and Wu (1999) from Olweus’ (1996) original BVQ. The subscale was administered only at T3, asking about bullying behaviors over the previous 6 months (roughly the entire last semester). The subscale comprises six items (e.g., deliberately hitting, kicking, pushing, or shoving others), rated on a 5-point scale (0 = never, 4 = five or more times), with higher scores indicating more frequent bullying. The Cronbach’*α* coefficient of the scale was 0.95.

### 2.3 Data analysis

Firstly, SPSS 27.0 was used to conduct the reliability analysis of these scales. Subsequently, descriptive and correlation analyses were performed for the study variables, and an independent samples t-test was conducted to examine sex differences. Then, a multiple mediation model analysis was performed using Mplus 8.3. With maltreatment as the independent variable, self-esteem and depression as mediating variables, and bullying behavior as the dependent variable, the bootstrap method was utilized with a sample estimate of 1,000 for the mediation model analysis. Finally, differences between genders in both direct and indirect effects were examined through multi-group analysis and Wald tests. Model fit was evaluated using the following criteria: *χ*^2^/df < 5, CFI and TLI > 0.90, RMSEA between 0.05 and 0.08, and SRMR < 0.08 (Hu and Bentler, 1999).

To investigate missing data patterns, the sample was first divided by school type (urban vs. rural). Little’s Missing Completely at Random (MCAR) test was conducted for each subsample and was found to be significant for both the rural school sample, *χ*^2^_(55)_ = 75.42, *p* = 0.035, and the urban school sample, *χ*^2^_(55)_ = 74.26, *p* = 0.043. These results indicated that the data were not missing completely at random. Subsequently, to assess the Missing at Random (MAR) assumption, we examined the relationship between missingness on key study variables and several auxiliary variables (i.e., age, subjective SES, and gender). As detailed in Supplementary Tables S1–S4, missing data in both subsamples were systematically related to these auxiliary variables, a pattern consistent with the MAR assumption. Therefore, Full Information Maximum Likelihood (FIML) was employed as a robust and appropriate method for handling the missing data. The formal analyses consequently included age, SES, and gender as auxiliary variables within the FIML estimation to ensure unbiased and robust results.

## 3 Results

### 3.1 Measurement invariance

As presented in Table 1, the measurement invariance of the CTQ, SES, CES-D, and BVQ scales was assessed across genders. Stepwise model comparisons revealed negligible changes in model fit, with differences in fit indices falling below established thresholds (ΔCFI < 0.01; ΔTLI < 0.01). These results establish that the scales achieved configural, metric, and scalar invariance across genders.

**Table 1:** Measurement invariance test by gender.

Models	*χ* ^2^	df	CFI	TLI	RMSEA	△CFI	△TLI	△RMSEA
Childhood Trauma Questionnaire (CTQ)								
M0 (Configural)	880.53	322	0.923	0.910	0.062	–	–	–
M1 (Metric)	917.45	338	0.921	0.911	0.062	−0.002	0.001	0.000
M2 (Scalar)	952.65	354	0.918	0.912	0.061	−0.003	0.001	−0.001
Self-Esteem Scale (SES)								
M0 (Configural)	67.75	58	0.994	0.991	0.018	–	–	–
M1 (Metric)	75.90	67	0.995	0.993	0.016	0.001	0.002	−0.002
M2 (Scalar)	94.23	76	0.989	0.987	0.022	−0.006	−0.006	0.006
Center for Epidemiological Studies Depression (CES-D) scale								
M0 (Configural)	713.08	320	0.917	0.901	0.054	–	–	–
M1 (Metric)	735.30	336	0.915	0.904	0.053	−0.002	0.003	−0.001
M2 (Scalar)	777.28	352	0.910	0.903	0.053	−0.005	−0.001	0.000
Bully/Victim Questionnaire (BVQ)								
M0 (Configural)	39.01	18	0.969	0.959	0.053	–	–	–
M1 (Metric)	41.20	23	0.973	0.965	0.043	0.004	0.006	0.010
M2 (Scalar)	49.25	28	0.969	0.967	0.042	−0.004	0.002	−0.001

### 3.2 Descriptive statistics and correlation analysis

Table 2 shows that the child emotional and physical maltreatment showed moderately significant negative correlations with self-esteem and moderately significant positive correlations with depression and bullying. Self-esteem had a moderately significant negative correlation with depression and bullying respectively, and depression had a moderately significant positive correlation with bullying. The sex differences of each variable were examined using independent samples t-tests, and the results showed that there were significant sex differences in emotional maltreatment at T1 (*t* = 2.44, *p* = 0.015, *Cohen’s d* = 0.19), physical maltreatment at T1 (*t* = 2.47, *p* = 0.014, *Cohen’s d* = 0.19), and bullying at T3 (*t* = 4.91, *p* < 0.001, *Cohen’s d* = 0.34). The scores of male students in emotional maltreatment at T1, physical maltreatment at T1 and bullying at T3 were significantly higher than those of female students. School location (e.g., urban vs. rural) was not significantly associated with any of the key variables in this study, such as bullying and depression (*p*s > 0.05), while age and subjective SES are significantly correlated with some of the key variables. Consequently, the subsequent model analysis controlled for age and subjective SES but excluded school location.

**Table 2:** Bivariate correlations and descriptive for study variables.

Variables	1	2	3	4	5	6	7	9
1. School location	1	0.02	0.06	−0.02	0.07	−0.04	0.04	0.03
2. T1 Age	0.02	1	−0.05	0.04	0.04	−0.04	0.14^**^	0.04
3. Subjective SES	0.01	−0.04	1	−0.09^*^	−0.06	0.05	−0.11^*^	−0.05
4. T1 Child Emotional Maltreatment	−0.05	−0.07	−0.05	1	0.69^**^	−0.33^**^	0.31^**^	0.23^**^
5. T1 Child Physical Maltreatment	−0.07	−0.10	−0.08	0.67^**^	1	−0.32^**^	0.40^**^	0.37^**^
6. T2 Self-Esteem	−0.03	0.02	0.001	−0.26^**^	−0.24^**^	1	−0.35^**^	−0.26^**^
7. T2 Depression	0.002	−0.22^**^	−0.04	0.35^**^	0.39^**^	−0.43^**^	1	0.30^**^
8. T3 Bullying	−0.09	0.001	−0.02	0.18^**^	0.24^**^	−0.17^**^	0.20^**^	1
M ± SD (The whole sample)				19.63 ± 8.39	20.52 ± 8.98	2.91 ± 0.43	22.65 ± 11.05	1.91 ± 4.34
M ± SD (Boys)				20.20 ± 8.41	21.15 ± 9.05	2.92 ± 0.45	22.67 ± 11.02	2.44 ± 4.84
M ± SD (Girls)				18.66 ± 8.28	19.47 ± 8.79	2.90 ± 0.41	22.61 ± 11.13	0.98 ± 3.09
*t*				2.44	2.47	0.80	0.06	4.91
*p*				0.015	0.014	0.437	0.953	<0.001
Cohen’s *d*				0.19	0.19	0.06	0.01	0.34

The above diagonal is male, below diagonal is female; M, mean; SD, Standard Deviation.

^**^p < 0.01, ^*^p < 0.05.

### 3.3 The mediating effects of self-esteem and depression between child maltreatment and bullying

We tested four competing models to examine the relationship between child maltreatment and bullying (Table 3): a direct-effects-only model (Model 0); single-mediator models for self-esteem (Model 1) and depression (Model 2); and a chain mediation model involving both self-esteem and depression (Model 3). Among these, the chain mediation model (Model 3) provided the best fit to the data, as indicated by the most favorable fit indices (i.e., the lowest *χ*^2^/df and the highest CFI and TLI). Model 3 shows that child emotional maltreatment (T1) does not predict child bullying (T3) when mediating variables are taken into account (*β* = −0.09, *p* = 0.109), while child physical maltreatment (T1) significantly and positively predicted child bullying (T3) (*β* = 0.29, *p* < 0.001). Self-esteem (T2) was negatively predicted by child emotional maltreatment (T1) (*β* = −0.21, *p* < 0.001), but depression (T2) was not predicted by child emotional maltreatment (T1) (*β* = 0.07, *p* = 0.235). Physical maltreatment in childhood (T1) negatively predicted self-esteem (T2) (*β* = −0.14, *p* = 0.006) and positively predicted depression (T2) (*β* = 0.27, *p* < 0.001). Depression (T2) was also significantly and negatively predicted by levels of self-esteem (T2) (*β* = −0.29, *p* < 0.001).

**Table 3:** Comparison of path coefficients and fit indices for competing mediation models.

Variables	Model 0: Bul (T3)		Model 1: Bul (T3)		Model 2: Bul (T3)		Model 3: Bul (T3)	
	β	SE	β	SE	β	SE	β	SE
Age	0.03	0.04	0.03	0.04	0.03	0.04	0.03	0.04
Sex	−0.13^***^	0.03	−0.14^***^	0.03	−0.13^***^	0.03	−0.14^***^	0.03
Subjective SES	−0.02	0.01	−0.02	0.01	−0.01	0.01	−0.01	0.01
CEM (T1)	−0.04	0.05	−0.08	0.06	−0.06	0.06	−0.09	0.06
CPM (T1)	0.35^***^	0.06	0.33^***^	0.06	0.29^***^	0.06	0.29^***^	0.06
S-E (T2)			−0.16^***^	0.04			−0.12^**^	0.04
Dep (T2)					0.18^***^	0.05	0.13^**^	0.05
*χ* ^2^	12.69		16.5		16.11		19.15	
*df*	6		9		9		12	
CFI	0.93		0.96		0.97		0.98	
TLI	0.94		0.95		0.96		0.97	
RMSEA	0.04		0.03		0.03		0.03	
SRMR	0.04		0.03		0.04		0.03	

CEM, Child Emotional Maltreatment; CPM, Child Physical Maltreatment; S-E, Self-esteem; Dep, Depression; Bul, Bullying; ^*^p < 0.05, ^**^p < 0.01.

To test the mediating effects, we used the deviation-corrected percentile bootstrap method (repeated sampling 1,000 times). In Table 4, child emotional maltreatment (T1) and child bullying (T3) were significantly mediated by self-esteem (T2) (*β* = 0.013, *p* = 0.027, 95% CI [0.004, 0.029]), and child physical maltreatment (T1) and child bullying (T3) were significantly mediated by depression (T2) (*β* = 0.018, *p* = 0.020, 95% CI [0.005, 0.036]). The chain mediating effects of self-esteem (T2) and depression (T2) in the influence pathway of child emotional maltreatment (T1) on child bullying (T3), was very low (*β* = 0.004, 95% CI [−0.000, 0.010]) and not significant (*p* = 0.068). Similarly, self-esteem (T2) and depression (T2) in the influence pathway of child physical maltreatment (T1) on child bullying (T3), the effect size was very low (*β* = 0.003, 95% CI [−0.000, 0.007]) and not significant (*p* = 0.077). Therefore, we did not discuss the chain mediating effect.

**Table 4:** Standardized coefficients and confidence intervals of indirect pathways.

Regression model path	Total sample			Boys			Girls			Wald test
	*β*	SE	95% CI	*β*	SE	95% CI	*β*	SE	95% CI	
CEM (T1) → S-E (T2) → Bul (T3)	0.013^*^	0.006	[0.004, 0.029]	0.017^*^	0.008	[0.002, 0.032]	0.008	0.006	[−0.004, 0.020]	0.857
CPM (T1) → S-E (T2) → Bul (T3)	0.008	0.004	[0.002, 0.021]	0.011	0.006	[−0.000, 0.230]	0.004	0.004	[−0.004, 0.013]	0.856
CEM (T1) → Dep (T2) → Bul (T3)	0.005	0.005	[−0.002, 0.018]	0.003	0.007	[−0.010, 0.016]	0.003	0.005	[−0.007, 0.014]	0.003
CPM (T1) → Dep (T2) → Bul (T3)	0.018^*^	0.008	[0.005, 0.036]	0.029^*^	0.013	[0.005, 0.053]	0.005	0.007	[−0.009, 0.018]	2.979
CEM (T1) → S-E (T2) → Dep (T2) → Bul (T3)	0.004	0.002	[−0.000, 0.010]	0.005	0.003	[−0.000, 0.011]	0.002	0.002	[−0.003, 0.006]	1.124
CPM (T1) → S-E (T2) → Dep (T2) → Bul (T3)	0.003	0.001	[−0.000, 0.007]	0.004	0.002	[−0.000, 0.007]	0.001	0.001	[−0.002, 0.004]	1.137

CEM, Child Emotional Maltreatment; CPM, Child Physical Maltreatment; S-E, Self-esteem; Dep, Depression; Bul, Bullying.

^**^p < 0.01, ^*^p < 0.05.

### 3.4 Testing for the sex difference

**Figure 2:**
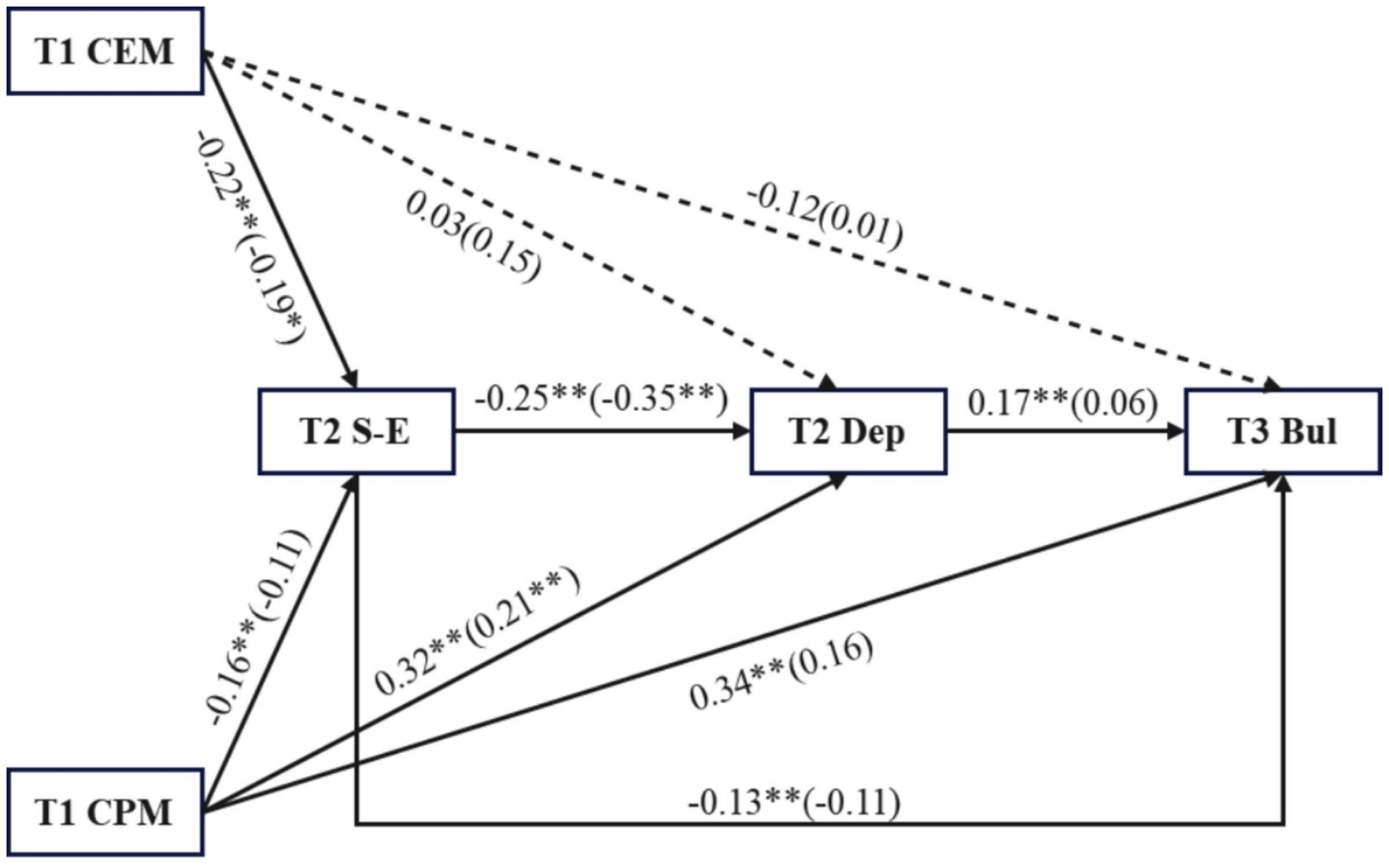
Sex difference in longitudinal dual-process model. The standardized coefficients were in all paths and girls’ results in brackets, controlling for age and subjective SES. CEM, Child Emotional Maltreatment; CPM, Child Physical Maltreatment; S-E, Self-esteem; Dep, Depression; Bul, Bullying; ^*^p < 0.05, ^**^ *p* < 0.01.

To compare sex differences, multiple group analyses were conducted. In the restricted model (all regression paths within the model were constrained to be equal across the male and female groups), the model fit was moderate (*χ*^2^ = 56.26, *df* = 25, RMSEA = 0.06, CFI = 0.91, TLI = 0.89, SRMR = 0.07), and in the unrestricted model, the model fit was excellent (*χ*^2^ = 30.89, *df* = 16, RMSEA = 0.05, CFI = 0.96, TLI = 0.92, SRMR = 0.05), △CFI = 0.05, △TLI = 0.03, indicating significant differences between the restricted and unrestricted models, and it is necessary to test the mediating model separately by sex. As shown in Figure 2, the paths from T1CPA to T2S-E, T1CPA to T3Bul, T2S-E to T3bul, and T2Dep to T3 Bul were significant in the male group but not in the female group. *Wald* test revealed a significant sex difference in the path from T2S-E to T2Dep, with a larger coefficient in the female group. The indirect effect analysis in Table 4 showed that in the male group, the indirect effect of T1 child-emotional-maltreatment through T2 self-esteem on T3 bullying was significant (effect size = 0.017, SE = 0.008, 95% CI [0.002, 0.032]), and the indirect effect of T1 child-physical-maltreatment through T2 depression on T3 bullying was significant (effect size = 0.029, SE = 0.013, 95% CI [0.005, 0.053]), while the indirect effects of the other paths were not significant. In the female group, all the indirect effects were not significant.

## 4 Discussion

This longitudinal study examined the mechanisms through which child maltreatment influences bullying perpetration among Chinese children, with particular attention to the mediating roles of self-esteem and depression, as well as gender-specific pathways. The findings not only corroborate but also extend existing theoretical frameworks while yielding culturally-specific implications for intervention.

### 4.1 Child maltreatment and bullying

The present study found that both physical maltreatment and emotional maltreatment significantly predicted subsequent bullying behavior, supporting Hypothesis 1. Building on Li et al.’s (2025) longitudinal work on the link between childhood maltreatment and bullying, our findings further clarify the unique predictive effects of these two forms of maltreatment. First, the association between physical maltreatment and bullying aligns with social learning theory (Burton et al., 2002), which emphasizes that observational learning is a key mechanism in behavioral acquisition (Giesen and Frings, 2021; Ramsey et al., 2021). In families where physical maltreatment occurs, perpetrators provide children with vivid and direct models of aggression through acts such as hitting or shoving (Pyburn, 2017; Li et al., 2021). Children who repeatedly witness the use of physical maltreatment to gain power or release emotion may later imitate these behaviors, transferring the pattern of using violence to achieve control or gratification into school settings, thereby developing bullying behaviors (Davis et al., 2020). However, this study also found that self-esteem fully mediated the relationship between emotional maltreatment and bullying, indicating that emotional maltreatment predicts bullying entirely through its impact on self-esteem. This suggests that the above explanation may be limited when applied to emotional maltreatment, as the mechanism linking emotional maltreatment to bullying differs from that of physical maltreatment. This difference likely stems from the distinct “content” learned through each type of maltreatment: physical maltreatment teaches children overt aggressive behaviors (Ran et al., 2023), whereas emotional maltreatment—through degradation, humiliation, and ridicule—instills negative beliefs about the self, leading children to internalize thoughts such as “I am bad” or “I am unlovable” (Melamed et al., 2024; Talmon et al., 2021). Consequently, the motivations behind bullying behaviors also differ. For individuals who experienced physical maltreatment, bullying tends to become a habitual tool for problem-solving or goal attainment, with alleviating low self-esteem being only an occasional byproduct (Lin et al., 2025). In contrast, for victims of emotional maltreatment, bullying may serve as an outlet for the pain accumulated from chronic low self-esteem and as a means to gain compensatory feelings of power or superiority (Bai et al., 2025). Overall, our findings refine the cycle-of-violence hypothesis (Widom, 1989) by highlighting that physical and emotional maltreatment influence bullying through distinct psychological mechanisms.

### 4.2 Mediation of self-esteem

Building on Hamstra and Fitzgerald’s (2022) longitudinal examination of the mechanisms linking childhood maltreatment to bullying via anxiety and social functioning, the present study extends this line of inquiry by foregrounding self-esteem as a unique mediator between emotional maltreatment and subsequent bullying. We found that emotional maltreatment indirectly elevates the likelihood of bullying perpetration by eroding self-esteem, partially supporting Hypothesis 2 and aligning with attachment theory’ s claim that early caregiver interactions are central to the formation of self-concept (Bowlby, 1969). Specifically, emotional maltreatment—conceptualized as a destructive early relational experience (Brown et al., 2026)—deprives children of positive feedback and emotional validation from primary caregivers, thereby undermining the development of a stable and positive sense of worth. The resultant fragile and unstable self-concept manifests as low self-esteem (Archuleta et al., 2024). Diminished self-esteem not only generates internal distress (Jiménez et al., 2017), motivating individuals to restore a transient sense of power and control through bullying as a form of psychological compensation, but also biases social-information processing. Heightened interpersonal sensitivity (Hardaker and Tsakanikos, 2022) increases the tendency to interpret ambiguous peer cues—an accidental bump or momentary neglect—as intentional provocation (Czajkowska-Łukasiewicz et al., 2025). This cognitive bias predisposes youth to adopt preemptive aggressive strategies, completing the mediated pathway from emotional maltreatment, through reduced self-esteem, to bullying behavior.

### 4.3 Mediation of depression

The present study also identified a partial mediating role of depression in the relation between physical maltreatment and bullying perpetration: physical maltreatment indirectly forecasts later bullying by intensifying depressive symptoms. This finding corroborates Hypothesis 3 and furnishes empirical support for both the hopelessness theory of depression (Rose and Abramson, 1992) and the behavioral catharsis model of aggression (Wolff and Ollendick, 2006). Specifically, children chronically exposed to physical maltreatment inhabit environments in which control over their own bodies is repeatedly usurped (Harris et al., 2022), fostering an “nothing I do can change my situation” attributional style that crystallizes into profound helplessness (Soral et al., 2021). Such helplessness is typically accompanied by intense negative affect and diminished self-efficacy (Smallheer and Dietrich, 2019)—core constituents of clinical depression (Ramos-Cejudo et al., 2024). To alleviate these aversive internal states, youth may externalize their distress by bullying others, redirecting intolerable depressive affect toward peers (Liu et al., 2022). Moreover, the social withdrawal and peer estrangement that characterize depressive states often consign youngsters to peripheral social positions (Dong et al., 2022), thereby intensifying their motivation to regain visibility or reassert dominance through coercive behavior (Pan et al., 2023). Together, these processes complete the developmental cascade from physical maltreatment, via depressive symptomatology, to bullying perpetration.

### 4.4 The chain mediating role of self-esteem and depression

The vulnerability model (Klein et al., 2011) proposes that maltreatment leads to bullying through a chain in which prolonged declines in self-esteem give rise to depression, the present longitudinal analysis did not detect this sequence. One likely reason is the relatively short span between measurement occasions: the three waves covered only 12 months. Aebi and Orth (2025), using continuous-time modeling across 17 years, found that the predictive effect of low self-esteem on depression peaks at approximately two years and explicitly recommended studying this effect over a multi-year horizon. Consequently, the current design may be insufficient to capture the cumulative process posited by the vulnerability model—namely, the gradual erosion of self-esteem that progressively intensifies depressive symptoms. Nevertheless, by disaggregating different forms of maltreatment, our study extends the vulnerability framework and underscores the need for more fine-grained longitudinal designs that can clarify the temporal dynamics involved.

### 4.5 The sex difference

In the male group, the indirect effect of T1 Child-Emotional-Maltreatment through T2 self-esteem on T3 bullying was significant, and the indirect effect of T1 Child-Physical-Maltreatment through T2 depression on T3 bullying was significant, while the indirect effects of the other paths were not significant. In the female group, all the indirect effects were not significant. Three cultural factors may explain the male-specific effects: (1) Upbringing styles: In China, sons are disciplined more harshly to cultivate “resilience” (Chen and Qin, 2020). (2) Sex roles: Traditional masculinity norms discourage emotional vulnerability, promoting aggressive coping (Eagly and Wood, 1999). (3) Societal expectations: Because of societal expectations of male, they may be reluctant to express emotions openly and instead use bullying as a way to mask their vulnerability. When facing depressive emotions, females tend to seek emotional support and professional assistance, such proactive coping strategies may reduce the likelihood of them responding through bullying behaviors. Of course, there is another possibility. The functional model of self-injury (Nock, 2010) indicates that self-injury behaviors can alleviate negative emotions such as depression. We speculate that abused women might trigger depression and subsequently turn the aggression inward, resulting in self-injury behaviors (Rahman et al., 2021). Future studies can delve deeper into this.

## 5 Limitations and implications

First, self-reported data were used in this study, which may be highly subjective, and the measurement process may be affected by other factors. Using multiple channels of data collection could be an option for future studies. Additionally, the validity of self-reported subjective SES among 8-year-old children may be limited due to their developmental stage and cognitive capacity to accurately perceive and report socioeconomic standing. This should be considered as an important measurement limitation. Second, this study only considered self-esteem and depression when exploring the indirect effects of child maltreatment on child bullying, and future researches could further consider other factors. Third, although our longitudinal design measured variables across three waves, it did not utilize a true longitudinal panel design with repeated measures of all constructs at each wave. Therefore, our analyses primarily model between-person differences. Future research would benefit from measuring all variables at each time point to more rigorously model within-person processes and change over time using methods such as cross-lagged panel models or latent growth curves. Fourth, the current study sample includes both children and adolescents, and the interpretation of the research results needs further validation in other samples. Finally, the bullying was measured only at the third time point (T3). This prevented us from examining the stability of bullying itself, i.e., its autoregressive effect. Consequently, the findings of this study should be interpreted with this constraint in mind.

This study explored the impact of child maltreatment from both forms (emotional and physical) on child bullying. On the one hand, emotional maltreatment could indirectly affect child bullying by affecting self-esteem. On the other hand, physical maltreatment could indirectly affect bullying in children by affecting depression. Furthermore, the damage from child maltreatment (emotional and physical) could last for a long time. This inspires us to pay attention to child maltreatment and consider the different impacts that different forms of child maltreatment may have on children. In the process of children’s growth, we should not only avoid corporal punishment of them, but also provide them with sufficient warmth and emotional support, thereby reducing their problematic behaviors and promoting their physical and mental health growth.

In practice, interventions tailored to specific types of maltreatment can be effectively implemented. For children experiencing emotional maltreatment, it is crucial to provide emotional support and psychological counseling aimed at enhancing self-esteem and emotional regulation skills. In the case of physical maltreatment victims, it is essential to offer physical protection and medical care, while simultaneously addressing their mental health to mitigate the risk of depression. At the policy level, the establishment of comprehensive child protection laws and systems is imperative. Strengthening the prevention and early identification of maltreatment, alongside the provision of integrated psychological services and social support networks, is necessary to foster a safer and healthier environment for children development.

## Data Availability

The original contributions presented in the study are included in the article/Supplementary material, further inquiries can be directed to the corresponding author.
