# Supplementary material for: Developmental pathway from child maltreatment to children’s bullying: sex difference in the longitudinal dual-process model of self-esteem and depression
Source: Front Psychol. 2025 Nov 7;16:1669772. doi: 10.3389/fpsyg.2025.1669772 (PMC12634508; doi:10.3389/fpsyg.2025.1669772)
Supplement: Supplementary file 1 [file Table_1.docx]

**Supplementary materials**

**Table S1**. Missing Pattern Analysis in Rural Schools

|  | | T1 age | Sub-SES | T1 CEM | T1 CPM | T2 Dep | T2 S-E | T3 Bul |
| --- | --- | --- | --- | --- | --- | --- | --- | --- |
| T1 CEM | t | -2.9 | .9 | — | -4.2 | -1.7 | 2.6 | -.9 |
|  | P | **.014** | .407 | — | **.001** | .109 | **.022** | .396 |
|  | Mean(Present) | 8.95 | 3.22 | 19.83 | 20.00 | 22.37 | 2.95 | 1.93 |
|  | Mean(Missing) | 9.37 | 3.08 | — | 28.42 | 28.18 | 2.69 | 3.17 |
| T1 CPM | t | .2 | -1.1 | -2.8 | — | -2.3 | .0 | .4 |
|  | P(2-tail) | .835 | .282 | **.011** | — | **.034** | .975 | .727 |
|  | Mean(Present) | 8.97 | 3.20 | 19.54 | 20.31 | 22.20 | 2.94 | 2.00 |
|  | Mean(Missing) | 8.95 | 3.39 | 24.94 | — | 28.94 | 2.94 | 1.67 |
| T2 Dep | t | -.1 | 1.2 | -3.1 | -2.1 | — | 1.9 | -2.0 |
|  | P(2-tail) | .919 | .248 | **.002** | **.036** | — | **.053** | **.053** |
|  | Mean(Present) | 8.97 | 3.24 | 18.93 | 19.70 | 22.62 | 2.96 | 1.65 |
|  | Mean(Missing) | 8.97 | 3.13 | 22.26 | 21.94 | — | 2.86 | 2.95 |
| T2 S-E | t | .6 | .5 | -.5 | -2.0 | -1.5 | — | 1.1 |
|  | P(2-tail) | .570 | .628 | .646 | .083 | .218 | — | .290 |
|  | Mean(Present) | 8.97 | 3.21 | 19.79 | 20.13 | 22.46 | 2.94 | 2.00 |
|  | Mean(Missing) | 8.88 | 3.11 | 21.33 | 26.56 | 31.00 | — | 1.25 |
| T3 Bul | t | .5 | .2 | -.9 | -.1 | .8 | .3 | — |
|  | P(2-tail) | .587 | .805 | .392 | .914 | .433 | .796 | — |
|  | Mean(Present) | 8.97 | 3.21 | 19.69 | 20.29 | 22.79 | 2.94 | 1.99 |
|  | Mean(Missing) | 8.92 | 3.19 | 21.13 | 20.47 | 20.81 | 2.92 | — |

*Note:* CEM = child emotional maltreatment, CPM = child physical maltreatment, S-E= self-esteem, Dep = depression, Bul=Bullying.

**Table S2**. Missing Pattern Analysis in Urban Schools

|  | | T1 age | Sub-SES | T1 CEM | T1 CPM | T2 Dep | T2 S-E | T3 Bul |
| --- | --- | --- | --- | --- | --- | --- | --- | --- |
| T1 CEM | t | .4 | -.7 | — | -2.2 | -2.9 | .2 | -.2 |
|  | P(2-tail) | .698 | .473 | — | **.036** | **.009** | .816 | .813 |
|  | Mean(Present) | 9.06 | 4.11 | 19.47 | 20.45 | 22.32 | 2.89 | 1.84 |
|  | Mean(Missing) | 9.02 | 7.32 | — | 24.91 | 29.24 | 2.88 | 2.05 |
| T1 CPM | t | .5 | 1.4 | -3.1 | — | -.8 | .5 | -.4 |
|  | P(2-tail) | .609 | .160 | **.006** | — | .461 | .644 | .667 |
|  | Mean(Present) | 9.06 | 4.31 | 19.21 | 20.69 | 22.60 | 2.90 | 1.83 |
|  | Mean(Missing) | 8.99 | 3.53 | 24.90 | — | 23.88 | 2.86 | 2.28 |
| T2 Dep | t | .8 | -.9 | -3.0 | -3.1 | — | 2.9 | -2.1 |
|  | P(2-tail) | .453 | .392 | **.003** | **.002** | — | **.004** | **.034** |
|  | Mean(Present) | 9.07 | 3.99 | 18.81 | 19.96 | 22.66 | 2.93 | 1.56 |
|  | Mean(Missing) | 9.02 | 5.38 | 22.08 | 23.47 | — | 2.76 | 2.93 |
| T2 S-E | t | .0 | 1.0 | -.8 | .9 | — | — | -.5 |
|  | P(2-tail) | .992 | .319 | .519 | .459 | — | — | .668 |
|  | Mean(Present) | 9.06 | 4.28 | 19.43 | 20.71 | 22.66 | 2.89 | 1.84 |
|  | Mean(Missing) | 9.06 | 3.67 | 24.67 | 18.33 | 25.00 | — | 3.67 |
| T3 Bul | t | -.1 | 2.4 | -.1 | .3 | -.9 | .7 | — |
|  | P(2-tail) | .920 | **.017** | .907 | .775 | .357 | .479 | — |
|  | Mean(Present) | 9.06 | 4.40 | 19.45 | 20.73 | 22.48 | 2.90 | 1.85 |
|  | Mean(Missing) | 9.07 | 3.07 | 19.60 | 20.34 | 24.22 | 2.85 | — |

*Note:* CEM = child emotional maltreatment, CPM = child physical maltreatment, S-E= self-esteem, Dep = depression, Bul=Bullying.

**Table S3**. Analysis of Gender Differences in Missing Patterns in Rural Schools

|  |  | Boys | Girls | χ^2^ | P |
| --- | --- | --- | --- | --- | --- |
| T1 CEM | Present | 204 | 130 | 0.949 | 0.330 |
|  | Missing | 9 | 3 |  |  |
| T1 CPM | Present | 204 | 124 | 1.072 | 0.300 |
|  | Missing | 9 | 9 |  |  |
| T2 Dep | Present | 157 | 98 | 0.000 | 0.996 |
|  | Missing | 56 | 35 |  |  |
| T2 S-E | Present | 207 | 130 | 0.102 | 0.750 |
|  | Missing | 6 | 3 |  |  |
| T3 Bul | Present | 197 | 117 | 1.992 | 0.158 |
|  | Missing | 16 | 16 |  |  |

*Note:* CEM = child emotional maltreatment, CPM = child physical maltreatment, S-E= self-esteem, Dep = depression, Bul=Bullying.

**Table S4**. Analysis of Gender Differences in Missing Patterns in Urban Schools

|  |  | Boys | Girls | χ^2^ | p |
| --- | --- | --- | --- | --- | --- |
| T1 CEM | Present | 265 | 147 | 4.918 | 0.027 |
|  | Missing | 9 | 13 |  |  |
| T1 CPM | Present | 263 | 152 | 0.234 | 0.628 |
|  | Missing | 11 | 8 |  |  |
| T2 Dep | Present | 215 | 131 | 0.726 | 0.394 |
|  | Missing | 59 | 29 |  |  |
| T2 S-E | Present | 272 | 159 | 0.016 | 0.899 |
|  | Missing | 2 | 1 |  |  |
| T3 Bul | Present | 252 | 140 | 2.310 | 0.129 |
|  | Missing | 22 | 20 |  |  |

*Note:* CEM = child emotional maltreatment, CPM = child physical maltreatment, S-E= self-esteem, Dep = depression, Bul=Bullying.
